# Effects of Wrist Stability Training Combined with Grip Strength Exercise on Pain and Function in Patients with Nonspecific Chronic Wrist Pain

**DOI:** 10.3390/medicina60071144

**Published:** 2024-07-16

**Authors:** Seung-Ji Hong, Mi-Young Lee, Byoung-Hee Lee

**Affiliations:** 1 Graduate School of Physical Therapy, Sahmyook University, Seoul 01795, Republic of Korea; ghdtmdwl8172@naver.com; 2 Department of Physical Therapy, Sahmyook University, Seoul 01795, Republic of Korea; mylee@syu.ac.kr

**Keywords:** wrist pain, grip strength, wrist function

## Abstract

*Background and Objectives:* Non-specific chronic wrist pain is wrist pain that occurs without a specific cause, such as trauma, and may limit the range of motion of the joints of the wrist and hand, affecting muscle strength, grip strength, and function. This study aimed to determine the effects of grip-strengthening exercises combined with wrist stability training on pain and function in patients with non-specific chronic wrist pain. *Materials and Methods:* The subjects of the study were 31 patients with wrist pain. To determine the effect of grip-strengthening exercises combined with wrist stability training, 15 participants participated in grip-strengthening exercises combined with wrist stability training and 16 control subjects participated. The experimental group participated in wrist-stability training. Grip-strengthening exercises combined with wrist stability training were performed for 20 min/day twice a week for 4 weeks, and relaxation massage and conservative physical therapy were performed for 20 min/day twice a week for 4 weeks. The control group received relaxation massage and conservative physical therapy for 40 min/day twice a week for 4 weeks. A visual pain scale was used to evaluate the degree of pain before and after treatment, and a patient-rated wrist evaluation was used to evaluate wrist function. *Results:* The results showed that the visual score significantly decreased in the time effect before and after the intervention in both groups (*p* < 0.001), patient-rated wrist evaluation significantly decreased (*p* < 0.001), and grip strength and muscle strength significantly increased (*p* < 0.001). The results of this study showed that grip-strengthening exercises combined with wrist stability training were effective in improving pain, function, grip strength, and muscle strength in patients with non-specific chronic wrist pain. *Conclusions:* Grip-strengthening exercises combined with wrist stability training can be used as an effective intervention method to improve pain, function, grip strength, and muscle strength, emphasizing the need for wrist exercise interventions in patients with non-specific chronic wrist pain in the future.

## 1. Introduction

In modern society, with the predominant use of computers, repetitive wrist strain in daily life is becoming more prevalent, leading to an increase in wrist pain [1]. The wrist, a bilateral joint placed in various positions during functional activities [2], can experience pain due to exposure to various types of stress, such as load pressure, twisting, and pulling [3].

Specific wrist pain can be exemplified by pain resulting from traumatic stress, fractures, inflammation of ligaments or tendons, or similar conditions. Conversely, non-specific wrist pain refers to general pain that does not originate from specific anatomical structures and is not caused by any particular activity [4]. For instance, non-specific chronic wrist pain is commonly observed in individuals who use their wrists extensively and are subjected to stress during daily activities, such as musicians, athletes, physical education teachers, cheerleaders, and drivers. Currently, there is an increase in patients with non-specific wrist pain due to various sports or leisure activities and excessive work demands [5]. Wrist movements include flexion and extension, radial deviation, ulnar deviation, pronation, and supination. To achieve mobility, a controlled system of ligaments, muscles, and tendons with relative stability is required [1]. Typically, limitations in the range of motion of the wrist and hand joints can be caused by wrist pain, swelling, muscle weakness, or disability factors and may lead to decreased grip strength, grasping ability, fine manipulation, and hand function [6]. Therefore, range of motion (ROM) measurement of the joints is an essential component of wrist joint assessment because it allows for the measurement of the degree of injury and evaluation of the functional status of the hand [7].

Patients with chronic wrist pain have been found to have decreased grip strength compared to those without wrist pain, indicating a correlation between wrist pain and grip strength [8]. Grip strength is influenced by various factors such as age, sex, body mass index, occupation, leisure activities, upper limb strength, nutritional status, pain, sensory loss, cognitive impairment, and others [9]. Furthermore, grip strength is generated by the co-contraction of the wrist flexor and extensor muscles, with activation of the extensor muscles playing a crucial role in stabilizing the wrist during gripping.

Strength training exercises can lead to isotonic strengthening of the wrist flexors and extensors [10]. Wrist stability training improves muscle balance and activation around the joint, providing stability to unstable joints, and stabilizing exercises effectively alleviate pain associated with musculoskeletal issues, such as low back pain and osteoarthritis [11]. Patients with chronic pain due to wrist instability are focused on pain reduction through wrist splinting [12], stabilizing exercises [10], and anti-inflammatory injections. Strengthening exercises through stabilizing movements can promote long-term wrist position control, strengthening, and functional stability enhancement [13].

This study aimed to investigate the effects of grip strength exercises combined with wrist stabilization training on pain and wrist function in patients with non-specific chronic wrist conditions. This study aimed to provide information on effective exercise methods for patients with chronic wrist pain.

## 2. Materials and Methods

### 2.1. Participants

This study included 34 adult patients with non-specific chronic wrist pain at G Hospital, Seoul, Republic of Korea. Before recruiting participants for this study, we performed a power analysis using G*Power version 3.1.9.7 (Heinrich-Heine Universität, Düsseldorf, Germany). Repeated measures ANOVA was performed and an effect size f of 0.25 was obtained for all the outcome measures. An α error probability of 0.05 was used in order to minimize the type 1-β error probability of 80%. The participants were divided into two groups and each subject received five measurements. Since the estimated target sample size was 32, we recruited 45 participants who had undergone physical therapy.

The selection criteria included patients in their 30s to 50s who had been experiencing wrist pain for over three months, those with wrist pain of unknown cause, and individuals capable of understanding and following the instructions provided by the researchers [14]. The exclusion criteria for the study participants included those experiencing wrist pain for less than three months, individuals aged 60 years or older, those with neurological or psychiatric disorders, individuals diagnosed with rheumatoid arthritis, and those who had undergone wrist surgery within the past six months [15].

All participants signed a consent form after the procedure, and the purpose of the study was explained. This study was approved by the Sahmyook University Institutional Review Board (approval number: SYU 2023-05-005-015) and Clinical Research Information Service (KCT0008988). Participants fully understood the objectives and procedures used in the study. The study adhered to the ethical principles of the Declaration of Helsinki.

### 2.2. Experimental Procedure

All mandatory record access approval was obtained before experimentation and the clinical characteristics of all study participants were collected, including medical history, present symptoms, surgical dates, and general characteristics, such as age, height, weight, and obesity status. In this study, 45 participants were recruited. After excluding those who were not in their 30s to 50s (*n* = 8) and those with a pain duration of less than three months (*n* = 3), 34 participants were divided into the experimental and control groups. To minimize errors related to group selection, the Research Randomizer program (http://www.randomizer.org/, accessed on 24 July 2023) was used to randomly divide the participants into two groups. This random assignment was performed to minimize bias and ensure that the experiment was conducted in an unbiased manner.

The experimental group underwent grip strength training combined with wrist stabilization exercises, wrist relaxation massage, and conservative therapy (infrared radiation + electrical stimulation + laser therapy) for 40 min per session twice a week, for four weeks. The control group received wrist relaxation massage and conservative therapy (infrared radiation + electrical stimulation + laser therapy) for 40 min per session twice a week, for four weeks. Pre- and post-assessments included the visual pain scale, wrist patient evaluation scale, grip strength, and muscle strength measurements. During the intervention, 2 participants in the experimental group dropped out due to health issues, and 1 participant in the control group dropped out, leaving a final total of 31 participants who completed the study.

The intervention was administered by physiotherapists with over five years of experience, while measurements were conducted by physiotherapists with over three years of experience. They were knowledgeable about the characteristics of non-specific chronic wrist pain and potential issues that could arise during the study process. Additionally, they received training on the use of measurement equipment one week before starting treatment to minimize error margins. 

Furthermore, measurements were conducted by the same physiotherapist before and after the experiment, and the assessor was blinded to the patient group.

### 2.3. Training Program

#### 2.3.1. Grip-Strengthening Exercises Combined with Wrist Stability Training (GSE Plus WST)

The participants in the experimental group had a therapist present beside them during grip strength training to ensure that they maintained the correct posture while seated and to provide any necessary adjustments.

During the exercise, a small table was placed in front of the participants to keep their elbows fixed while performing the exercise. The therapist demonstrated the exercise from the side to allow the patient to replicate it and provided repeated corrections to ensure that the exercises were performed correctly. Patients were instructed to discontinue treatment if pain increased during the exercise session compared with the initial pain assessment. The weight of the dumbbells and the resistance level of the elastic bands were adjusted according to the patient’s condition and tolerance, and adequate rest periods were provided between treatments. Exercise therapy was administered only on the affected side. The total exercise duration was 20 min, with a rest period of 30 s between sets (Table 1).

#### 2.3.2. Relaxation Massage

The study was conducted on subjects in both the experimental and control groups. Wrist relaxation massage was performed with the patient lying flat, the elbows extended, and the wrists in a neutral position.

The therapist primarily applied a wrist relaxation massage by pressing on the muscles around the wrist, including the wrist extensors, wrist flexors, abductor pollicis longus, and opponens pollicis, using the therapist’s thumb. The relaxation massage was conducted without rest periods and tailored to the patient’s condition. During the first and second weeks, the control group received a relaxation massage for 15 min, with pressure applied at a low intensity. During the first and second weeks, the experimental group received a relaxation massage for 10 min, with pressure applied at a low intensity. During the third and fourth week, the control group received a relaxation massage for 20 min, with pressure applied at a moderate intensity. During the third and fourth week, the experimental group received a relaxation massage for 10 min, with pressure applied at a moderate intensity.

#### 2.3.3. Conservative Physical Therapy

Both the experimental and control groups underwent these procedures, and conservative physical therapy was administered in the hospital’s physical therapy department. Conservative physical therapy consists of infrared, interferential current therapy, and laser therapies. During treatment, the patient remained in the supine position with the wrist in a neutral state, and treatment was administered to the area around the wrist joint. The intensity and treatment range of infrared therapy, interferential current therapy, and laser therapy were adjusted by the therapist according to the patient’s condition, and the treatments were administered without rest periods.

#### 2.3.4. Outcome Measures

To assess the level of pain in patients with non-specific chronic wrist pain, the Visual Analog Scale (VAS) was used, with reliability ranging from 0.60 to 0.77, according to Boonstra et al. [16]. The far left of the scale is labeled ‘0’, and the far right is labeled ‘10’, with ‘0’ indicating no pain and ‘10’ indicating the most severe pain experienced so far [17].

The Patient-Rated Wrist Evaluation (PRWE) questionnaire had a high reliability of 0.93, making it a reliable method for assessing wrist function, as reported by Bastard et al. [18]. The measurement method involved a questionnaire divided into scales ranging from 0 to 10 with distinct sections for pain and function. The questionnaire is comprised of 15 items, with scores ranging from 0 to 10 for each item, representing the patient’s perception of each aspect. The pain items approach ‘10’ as wrist pain increases, whereas the functional items approach ‘10’ as the difficulty in performing tasks increases. The total number of items was calculated, and the sum of the two functional items was divided into two. Finally, the total pain and adjusted functional total scores were summed. The maximum total score was 100 points. A lower total score indicated less pain and better wrist function.

The grip strength was measured using a digital device (Lavisen Co., Ltd., Namyangju, Republic of Korea, 2020). The participants were instructed to stand upright with their feet shoulder-width apart, elbows flexed, and forearms in a neutral position against their sides, and to grip the grip strength measurement device with maximum force for 2 s. After the practice session, grip strength was measured thrice, and the average value of these measurements was recorded as the grip strength value. A 30-s rest period was provided between each measurement. During each measurement, the therapist provided guidance to ensure that the participants exerted the maximum force as reported by Yu et al. [19].

Muscle strength was measured using a digital muscle strength measurement device, specifically the Commander Powertrack II Dynamometer, manufactured by J-Tech Medical in Midvale, UT, USA in 2011. Its reliability has been reported to be 0.82, according to Park et al. [20]. The unit of force used was Newtons (N). For each muscle group, muscle strength was measured for 5 s and the maximum value obtained during this period was recorded as the measurement value. 

Before the measurement, the participants received pre-measurement education and practiced each measurement movement. A 30-s rest period was provided between each measurement. For the muscle strength measurements, the participants were seated with their forearms fixed on a table to ensure that their wrists and hands were positioned outward without touching the table. During the assessment of wrist flexor muscle strength, the experimental participants were in a wrist flexion position with the wrist, whereas during the measurement of wrist extensor muscle strength, they were in a wrist extension position with the wrist facing downwards. To measure the radial and ulnar deviation muscle strength, the participants’ forearms were fixed on a table, ensuring that their wrists and hands were positioned outward without touching the table. The experimental subjects resisted the therapist’s resistance for 2 s and then rested for 30 s. This process was repeated three times and the average measurement was recorded.

#### 2.3.5. Data Analysis

Statistical analyses were conducted using SPSS (Statistical Package for the Social Sciences) 23.0 for Windows software. Descriptive statistics were used to analyze the general characteristics of the participants and examine the homogeneity between each group, and an independent *t*-test was conducted. Within-group differences before and after the intervention were analyzed using a paired *t*-test, whereas independent *t*-tests were used to compare the differences between the groups. Two-Way Analysis of Variance (Two-Way ANOVA) was used to assess the interaction between group and time. The statistical significance level for all data was set at *p* < 0.05. 

## 3. Results

### 3.1. General Characteristics of Participants

The subjects of this study were comprised of 15 participants in the wrist stability training combined with the grip-strengthening exercise group and 16 participants in the control group. All general characteristics and dependent variables followed a normal distribution, and homogeneity testing showed no significant differences between the groups in the pre-assessment results for all dependent variables (Table 2).

### 3.2. Changes in Pain, Wrist Function and Grip Strength

On the visual pain scale, the experimental group significantly decreased from 4.23 points before treatment to 1.87 points after treatment (*p* < 0.001), whereas the control group significantly decreased from 3.99 points to 2.67 points after treatment (*p* < 0.01). 

For wrist function, the experimental group significantly decreased from 39.33 points before treatment to 19.87 points after treatment (*p* < 0.001), while the control group also significantly decreased from 36.94 points to 27.78 points after treatment (*p* < 0.01). 

For grip strength, the experimental group significantly increased from 27.01 kg before treatment to 35.40 kg after treatment (*p* < 0.001), while the control group showed no statistically significant difference, decreasing from 28.53 kg to 28.43 kg after treatment.

Significant differences in pain, wrist function, and grip strength were observed over time (*p* < 0.001). However, there were no significant differences for any of the items in terms of group effects. Regarding the time × group interaction effect, there were significant differences observed in pain, wrist function, and grip strength (*p* < 0.001) (Table 3).

### 3.3. Changes in Wrist Muscle Strength

In wrist flexor muscle strength, the experimental group significantly increased from 55.03 N before treatment to 68.21 N after treatment (*p* < 0.001), while the control group increased from 53.65 N to 54.24 N after treatment, but there was no statistically significant difference. In wrist extensor muscle strength, the experimental group significantly increased from 60.75 N before treatment to 70.54 N after treatment (*p* < 0.001), while the control group increased from 57.25 N to 57.44 N after treatment, but there was no statistically significant difference. In wrist radial deviation muscle strength, the experimental group significantly increased from 56.90 N before treatment to 68.89 N after treatment (*p* < 0.001), while the control group increased from 50.87 N to 51.39 N after treatment, but there was no statistically significant difference. In wrist ulnar deviation muscle strength, the experimental group significantly increased from 60.29 N before treatment to 71.41 N after treatment (*p* < 0.001), while the control group decreased from 60.44 N to 58.13 N after treatment, with no statistically significant difference. 

There were significant differences in the wrist flexor, extensor, radial deviation, and ulnar deviation muscles over time (*p* < 0.01). In terms of group effects, there was a significant difference in the radial deviation muscles of the wrist (*p* < 0.05). Regarding the time × group interaction effect, significant differences were observed in the wrist flexor, extensor, radial, and ulnar deviation muscles (*p* < 0.001) (Table 4).

## 4. Discussion

Pain refers to unpleasant sensory and emotional experiences related to tissue damage or injury, serving the role of protecting the body from danger, and facilitating the healing of damaged tissue [21]. Many individuals with chronic pain experience severe ongoing pain, causing physical and emotional disabilities, as they often do not know how to alleviate pain [22].

This study was conducted to measure changes in pain through wrist stability training combined with grip-strengthening exercises. The visual pain scale scores had decreased from 4.23 points to 1.87 points, indicating a significant reduction in the visual pain scale within the group after treatment (*p* < 0.001). The effect of the time × group interaction showed significant differences in the experimental group (*p* < 0.001). In a study conducted by Sundstrup et al. [23], which investigated the effects of strength training on fatigue resistance and self-rated health in workers with chronic pain, 66 workers with chronic pain participated in the experiment. The results showed a significant reduction in pain from 2.4 to 0.8, representing a 41% decrease (*p* < 0.05). However, no statistically significant differences were observed between the groups. Galal et al. [15] studied the effects of high-power laser therapy combined with exercise on wrist pain, function, and joint position sense in female gymnasts with non-specific chronic wrist pain. The study involved 36 female gymnasts who were divided into three groups: laser therapy, exercise program, and combined therapy (laser and exercise therapy). All three groups showed differences within the groups (*p* < 0.05), and there were significant differences between the exercise program, laser therapy, and combined therapy groups (*p* < 0.05). However, no significant difference was observed between the exercise program and laser therapy groups.

Similar to previous studies, the present study confirmed the significant differences in pain. Previous studies that used strength training and proprioceptive exercises did not show significant differences in pain between the groups in group comparisons. However, in this study, a significant improvement in the visual pain scale scores was observed in the time × group interaction effect. Wrist joint pain can be associated with muscle laxity on the dorsal or palmar aspect of the wrist and can induce pain due to joint instability caused by imbalances in the surrounding soft tissues, such as the tendons [24]. 

Such instability can cause discomfort and interfere with wrist movements, thereby impairing the quality of life in daily activities and routine tasks. Ultimately, in this study, the grip-strengthening exercises combined with wrist stability training-induced increased muscle strength around the wrist by performing resistance and proprioceptive exercises using small equipment, thereby correcting imbalances in the muscles and tendons around the wrist by improving muscle tension and enhancing wrist joint mobility, thereby alleviating subjective pain levels and improving visual pain scale scores. The wrist joint is formed by the articulation of eight carpal bones and two forearm bones, and its movement is controlled by the muscles of the arm. Strong and short ligaments maintain the stability of the wrist [25].

The wrist is involved in several functional activities and is highly susceptible to trauma and degenerative diseases [26]. The wrist joint is a complex joint that allows movement in multiple directions, and its function affects the overall well-being and health status of patients [27]. In terms of wrist function, the grip-strengthening exercise combined with wrist stabilization training significantly decreased from 39.33 points to 19.87 points after treatment (*p* < 0.001) in this study. In addition, a significant difference was observed in the time × group interaction effect (*p* < 0.001).

In a previous study by Dorich and Cornwall [10], which compared the effects of grip-strengthening exercises in 32 patients with non-specific chronic wrist pain, significant differences were observed in wrist function after grip-strengthening exercises (*p* < 0.05). Abdelmegeed et al. [28] divided 30 subjects with scapular pain into three groups: a control group, a group using a scapular brace, and a group using a scapular brace along with strengthening exercises. When comparing the brace and brace plus strengthening exercises groups with the control group, significant differences were observed (*p* < 0.05). However, no significant differences were observed between the two experimental groups.

In this study, a significant difference was observed in the time × group interaction effect on wrist function improvement (*p* < 0.001). Jung et al. [11] reported that wrist stability training improves muscle activation, provides stability to unstable joints, and effectively alleviates pain associated with musculoskeletal problems such as osteoarthritis. Wrist exercises not only affect the muscles and tendons around the wrist joint but also strengthen the ligaments. Consequently, conducting grip-strengthening exercises combined with wrist stability training provides stability to the wrist and enhances its function more effectively than not exercising, thereby imparting a sense of stability during daily wrist use. This intervention is believed to be more effective in improving wrist function.

In this study, there was a significant increase in grip strength in the grip-strengthening exercise group combined with wrist stability training, which increased from 27.01 kg to 35.40 kg after treatment (*p* < 0.05). After treatment in the control group, grip strength decreased from 28.53 kg to 28.43 kg after treatment, with no significant difference. A significant difference was observed in the time × group interaction (*p* < 0.001). Dorich and Cornwall [10] found a significant difference in grip strength in their study examining the effects of a grip-strengthening algorithm in 32 patients with chronic wrist pain, with grip strength increasing from 32.2 lb to 47.9 lb (*p* < 0.05). In this study, adults with wrist pain participated and grip exercises were combined with wrist stability training, which gradually increased the exercise intensity. This approach likely induced an overall strengthening of the muscles around the wrist, leading to the observed differences in the time × group interaction effect. In this study, changes in wrist muscle strength were significant after grip-strengthening exercises combined with wrist stability training. Specifically, the wrist flexor muscle strength increased from 55.03 N to 68.21 N, the wrist extensor muscle strength increased from 60.75 N to 70.54 N, the radial deviation muscle strength increased from 56.90 N to 68.89 N, and the ulnar deviation muscle strength increased from 60.29 N to 71.41 N after treatment (*p* < 0.001). Regarding the time × group interaction effect, there were significant differences in the strengths of the wrist flexor, wrist extensor, radial deviation, and ulnar deviation muscles (*p* < 0.001).

Sundstrup et al. [23] studied the effects of strength training on fatigue resistance and self-assessed health in 66 workers with chronic pain. The experimental group experienced an 11% increase in strength (*p* < 0.05), while the control group showed a decrease in strength. The research findings indicated that similar to previous studies, there was a significant difference in the time effect. However, in terms of the group effect, only the ulnar deviation muscle strength of the wrist showed a significant increase (*p* < 0.05). As in previous studies, various exercises for strength improvement were implemented in this study. While significant increases were observed in all muscle strengths in the time effect and time × group interaction effect, achieving significant strength improvement in patients compared with the control group was challenging owing to the short duration and limited exercise time.

This study has several limitations that should be considered when interpreting the results. This study had a small sample size of 31 participants, which limited the generalizability of the findings. Additionally, wrist pain was observed not only in the dominant hand but also to some extent in the non-dominant hand. Conducting the experiment in a small clinic in a single region further restricts the generalizability of the results because of the short duration of the study, which lasted only four weeks. Therefore, there was insufficient time to demonstrate the effects of long-term exercise interventions. Additionally, when evaluating wrist function, some items in the assessment questionnaire involved activities that the participants may not have previously attempted, making it difficult to provide accurate responses. Another limitation was the difficulty in precisely determining which treatment was effective, owing to various treatment prescriptions within the hospital setting. Considering these limitations into account, recruiting a larger number of participants and establishing a long-term experimental intervention plan is necessary. Restricting other prescribed medications or treatments outside of the experimental intervention and conducting the study in multiple regions would further validate the effectiveness of wrist stability training combined with grip-strengthening exercises.

## 5. Conclusions

This study aimed to investigate the effects of wrist stability training combined with grip-strengthening exercises on pain, range of motion, and function in patients with nonspecific chronic wrist pain. Both groups underwent relaxation massage and conservative physical therapy sessions lasting 20–40 min, twice a week, for four weeks. Additionally, wrist stability training combined with grip-strengthening exercises was conducted for 20 min, twice a week, over the same four-week period. Significant differences were observed in pain, range of motion, function, grip strength, and muscle strength before and after the intervention. This study demonstrated that grip-strengthening exercises combined with wrist stabilization training have beneficial effects on pain, wrist function, grip strength, and muscle strength in patients with non-specific chronic wrist pain. In future clinical practice, wrist exercise intervention should be emphasized as a therapeutic approach for improving pain, function, grip strength, and muscle strength in patients with non-specific chronic wrist pain. Wrist stabilization training combined with grip-strengthening exercises applied in this study can be proposed as an effective intervention method.

## Figures and Tables

**Table 1:** Grip-strengthening exercises combined with wrist stability training.

Exercise	GSE Plus WST	Time
Grip the resistance ball with your entire hand	-Keep your elbows fixed on the table at a 90-degree angle, with a neutral wrist posture. -Hold the ball in your hand for 1 s, then squeeze, and release for 1 s.	10 repetitions, 2 sets, rest for 3 min
Grip the resistance ball with each finger individually	-Keep your elbows fixed on the table at a 90-degree angle, with a neutral wrist posture. -Hold the ball between your thumb and index finger, thumb and middle finger, thumb and ring finger, and thumb and little finger sequentially for 1 s each, then squeeze, and release for 1 s.	10 repetitions, 2 sets, rest for 6 min
Wrist resistance exercises using elastic bands	-Keep your elbows fixed on the table at a 90-degree angle. -While lying on your back, overcome the resistance of the band, perform wrist flexion, hold for 2 s, and return to neutral posture. -While lying face down, overcome the resistance of the band, perform wrist extension, hold for 2 s, and return to neutral posture.	Wrist flexion: 10 repetitions, 2 sets, rest for 3 min. Wrist extension: 10 repetitions, 2 sets, rest for 3 min
Wrist stabilization exercises using dumbbells	-Keep your elbows fixed on the table at a 90-degree angle, with a neutral wrist posture. -Hold dumbbells (0.5 kg–3 kg) and sustain for 30 s.	2 repetitions, 2 sets, rest for 3 min

**Table 2:** General characteristics of participants (*N* = 31).

Characteristics	GSE Group (*n* = 15)	Control Group (*n* = 16)	*t* (*p*)
Age (years)	39.73 (10.16) ^a^	37.43 (8.65)	0.678 (0.503)
Height (cm)	170.07 (7.93)	167.19 (6.42)	0.210 (0.274)
Weight (kg)	69.60 (12.40)	65.69 (11.63)	0.559 (0.372)
BMI (kg/m^2^)	23.88 (2.78)	23.47 (3.56)	0.358 (0.723)

^a^ M(SD); BMI = body mass index; GSE = Grip-strengthening exercises combined with wrist stability training.

**Table 3:** Comparison of pain (*N* = 31).

Parameters		GSE Group (*n* = 15)	Control Group (*n* = 16)	*t* (*p*)	Time *F* (*p*)	Group *F* (*p*)	Time × Group *F* (*p*)
VAS (score)	pre	4.23 (1.27) ^a^	3.99 (1.50)	0.479 (0.636) ^c^	225.593 (0.001)	0.424 (0.520)	17.961 (0.001)
	post	1.87 (0.86)	2.67 (1.27)				
	difference	2.37 (0.58)	1.33 (0.77)	4.238 (0.001)			
	*t* (*p*)	15.764 (0.001) ^b^	6.908 (0.001)				
PRWE (score)	pre	39.33 (14.30) ^a^	36.94 (13.18)	0.485 (0.631) ^c^	135.290 (0.001)	0.423 (0.520)	17.555 (0.001)
	post	19.87 (9.48)	27.78 (11.63)				
	difference	19.47 (8.11)	9.16 (5.41)	4.190 (0.001)			
	*t* (*p*)	9.299 (0.001) ^b^	6.767 (0.001)				
grip strength (kg)	pre	27.01 (9.72) ^a^	28.53 (13.14)	−0.363 (0.719) ^c^	54.438 (0.001)	0.436 (0.514)	57.013 (0.001)
	post	35.40 (11.02)	28.43 (12.10)				
	difference	8.39 (3.60)	−0.10 (2.61)	7.475 (0.001)			
	*t* (*p*)	−9.024 (0.001) ^b^	0.149 (0.884)				

^a^ M(SD); ^b^ Paired *t*-test; ^c^ Independent *t*-test; *F* = repeated measures ANOVA; GSE = Grip-strengthening exercises combined with wrist stability training; VAS = visual analogue scale; PRWE = patient-rated wrist evaluation.

**Table 4:** Comparison of wrist muscle strength (*N* = 31).

Parameters		GSE Group (*n* = 15)	Control Group (*n* = 16)	*t* (*p*)	Time *F* (*p*)	Group *F* (*p*)	Time × Group *F* (*p*)
Wrist Flexor Muscle (N)	pre	55.03 (14.58) ^a^	53.65 (20.76)	0.213 (0.833) ^c^	28.025 (0.001)	1.379 (0.250)	23.443 (0.001)
	post	68.21 (18.05)	54.24 (19.92)				
	difference	7.46 (15.14)	−3.01 (10.57)	4.842 (0.001)			
	*t* (*p*)	−5.437 (0.001) ^b^	−0.540 (0.597)				
Wrist Extensor Muscle (N)	pre	60.75 (18.80)	57.25 (21.44)	0.481 (0.634)	16.458 (0.001)	1.332 (0.258)	15.206 (0.001)
	post	70.54 (19.76)	57.44 (20.95)				
	difference	9.79 (7.93)	0.19 (5.65)	3.899 (0.001)			
	*t* (*p*)	−4.781 (0.001)	−0.137 (0.893)				
Wrist Radial Deviation Muscle (N)	pre	56.90 (16.59)	50.87 (17.43)	0.986 (0.332)	23.142 (0.001)	4.430 (0.044)	19.421 (0.001)
	post	68.89 (12.88)	51.39 (16.44)				
	difference	11.99 (8.74)	0.53 (5.48)	4.407 (0.001)			
	*t* (*p*)	−5.314 (0.001)	−0.383 (0.707)				
Wrist Ulnar Deviation Muscle (N)	pre	60.29 (13.92)	60.44 (26.06)	−0.019 (0.985)	9.199 (0.005)	0.882 (0.355)	21.407 (0.001)
	post	71.41 (13.37)	58.13 (22.46)				
	difference	10.71 (10.07)	−2.31 (6.23)	4.627 (0.001)			
	*t* (*p*)	−4.452 (0.001)	1.485 (0.158)				

^a^ M(SD); ^b^ Paired *t*-test; ^c^ Independent *t*-test; *F* = repeated measures ANOVA; GSE = Grip-strengthening exercises combined with wrist stability training.

## Data Availability

Data are contained within the article.
